# A review of international trade and investment agreements and nutrition policy space in the Pacific

**DOI:** 10.3389/fnut.2023.1208542

**Published:** 2023-08-30

**Authors:** Noah Bunkley, Judith McCool, Kelly Garton

**Affiliations:** Faculty of Medical and Health Sciences, The University of Auckland, Auckland, New Zealand

**Keywords:** Pacific, trade, investment, nutrition, policy space, trade liberalisation, TIA, SIDS

## Abstract

Small Island Developing States (SIDS) in the Pacific are heavily reliant on imported foods which are often nutritionally deficient, and typically high in salt, fat, and sugar. To curb nutrition-related non-communicable diseases, nutrition policies are needed to create food environments that promote healthy diets. However, international trade and investment agreements (TIAs) may interfere with the policy space for SIDS to regulate their food environments by requiring member states to meet trade obligations that could conflict with their nutrition policy goals. In this review, we identify real examples of where TIAs have been responsible for changes in Pacific SIDS’ nutrition policies alongside the potential for further constraints on healthy nutrition policies from Pacific Island participation in TIAs. In addition, we note the effects of regulatory chill from TIA obligations in Pacific SIDS, whereby healthy nutrition policies are not considered, developed, or implemented due to the threat of trade disputes or the complexity of TIA procedural requirements. Existing literature indicates that TIAs have shaped nutrition policies to fit within the global trade paradigm despite SIDS’ nutrition policy imperatives. More can be done locally, regionally, and internationally to increase the importance of nutrition in the trade agenda, leverage regional institutions to champion nutrition regulation and support SIDS in navigating the trade and nutrition policy environment.

## Introduction

Non-communicable diseases (NCDs) are responsible for 75% of deaths globally, of which a significant proportion of adult deaths are attributable to poor diets (1). NCDs are the single largest cause of premature death in the Pacific with a large proportion due to poor nutrition (2, 3). The rate of weight gain in the Pacific over the last several decades has been five times that of the rest of the world (3). In addition, SIDS are heavily reliant on international trade to meet their populations’ nutritional needs and much of this imported food is of poor nutritional quality and high in salt, fat and sugar (4). Pacific Island countries reported a 40% increase in processed food sales between 2004 and 2018 (5).

Healthy nutrition policies targeting the food environment (e.g., sugar-sweetened beverage (SSB) taxation, fruit and vegetable subsidies and front-of-package nutrition labelling) are recommended by the World Health Organization to combat the rising rates of nutrition-related NCDs in SIDS (6). Despite good evidence that these structural level changes are more likely to have a sustained impact, government attempts to introduce healthy nutrition policies are often strongly opposed by industry. International trade and investment agreements (TIAs) are one tool used by opposing stakeholders to challenge healthy legislation. TIAs can include multilateral, bilateral or regional free trade (and investment) agreements or investment treaties. When such international economic agreements interfere with domestic regulatory processes, this reduces the ‘space’ or freedom for policymakers to develop and implement healthy nutrition policies. Policy space is the ability of a government to pursue economic and social policies to achieve its own national goals (7). TIAs have been extensively documented as a potential barrier to governments’ policy space in the areas of nutrition, tobacco, and alcohol (8–12). For example, Phillip Morris International Inc.’s challenge to Australia’s tobacco plain packaging bill was a clear example of a direct industry attack to domestic health policies through trade and/or investment disputes (13).

TIAs typically privilege larger, high-GDP countries that are more likely to have resources to formulate and administer trade policy on favorable terms, finance dispute settlements, host representation in international trade forums, and navigate World Trade Organization (and other trade and investment governing bodies’) procedures and rules (14). Many Pacific SIDS undertake these negotiations with vastly fewer resources placing them at an unfair disadvantage within the global trade and investment landscape.

This review draws together literature on the impact of international TIAs on nutrition policies in SIDS in the Pacific. Six Pacific countries are members of the WTO, and there are several multilateral TIAs active in the region (Table 1) (15). We conducted a search of Medline (OVID), Scopus and PIAS databases in March 2022, including articles that identified or described factors involved in or instances where international trade and investment agreements, including bilateral, regional, and multilateral treaties, impacted nutrition policy space in the Pacific. We outline the theoretical grounding for TIAs limiting nutrition policy space, provide examples of real trade challenges impacting nutrition policy space and highlight several cases where TIAs have resulted in regulatory chill in the Pacific. We conclude by identifying several action areas for improving nutrition policy space in SIDS in the Pacific.

**Table 1:** Selected regional and plurilateral trade and investment agreements for small island developing states (SIDS) in the Pacific (15).

Date	Name	Countries	Type
1977	Australia – Papua New Guinea (PACTRA)	Australia; Papua New Guinea	Free Trade Agreement
1981	South Pacific Regional Trade and Economic Cooperation Agreement (SPARTECA)	Australia; Cook Islands; Fiji; Kiribati; Marshall Islands; Micronesia, Federated States of; Nauru; New Zealand; Niue; Papua New Guinea; Samoa; Solomon Islands; Tonga; Tuvalu; Vanuatu	Partial Scope Agreement
1994	Melanesian Spearhead Group (MSG)	Fiji; Papua New Guinea; Solomon Islands; Vanuatu	Partial Scope Agreement
2003	Pacific Island Countries Trade Agreement (PICTA)	Cook Islands; Fiji; Kiribati; Micronesia, Federated States of; Nauru; Niue; Papua New Guinea; Samoa; Solomon Islands; Tonga; Tuvalu; Vanuatu	Free Trade Agreement
2009	EU - Pacific States	European Union; Fiji; Papua New Guinea; Samoa; Solomon Islands	Free Trade Agreement
2020	Pacific Agreement on Closer Economic Relations Plus (PACER Plus)	Australia; Cook Islands; Kiribati; Nauru; New Zealand; Niue; Samoa; Solomon Islands; Tonga; Tuvalu; Vanuatu	Free Trade Agreement & Economic Integration Agreement
2021	United Kingdom - Pacific States	United Kingdom; Fiji; Papua New Guinea; Samoa; Solomon Islands	Free Trade Agreement

## Theoretical analyses of potential legal constraints arising from trade obligations

In the past decade scholars have debated the potential constraints arising from TIAs and WTO obligations for nutrition policy in the Pacific. The principles of non-discrimination, necessity and justification, market access requirements and quantitative restrictions, intellectual property rights and ‘fair and equitable’ treatment of investors may restrict the development and implementation of healthy nutrition policies that regulate food environments (16, 17). While few studies have directly assessed the potential legal constraints of TIAs on nutrition policy in SIDS, Ravuvu et al. noted several trade obligations in Fiji relating to potential restrictions of the government’s policy space (18). Fiji is not part of any bilateral trade agreement, yet it is subject to regional and WTO obligations, which could impact domestic policies regarding government procurement and enforcement, regulation of food marketing, and composition and labelling, as well as place Fiji at risk of dispute settlements in the event of obligation breaches (18).

Similarly, Vanuatu’s 2012 WTO accession package was found to have nine areas where nutrition policy space was potentially constrained (19). Some examples of these potential constraints included: (i) Vanuatu’s commitment to not implement any antidumping or safeguard measures until Vanuatu’s laws were consistent with WTO agreements, increasing the risk of more low-priced food imports; (ii) commitment not to undertake pre-shipment inspections of imports, potentially inhibiting the enforcement of food composition laws; and (iii) commitment to progressively liberalize its business environment by removing restrictions on foreign investments, risking increasing production and consumption of locally produced ultra-processed foods (19). The result of these concessions for Vanuatu is a restricted policy space particularly in the areas of regulating cheap, unhealthy imports and shaping local healthy food production.

In regard to nutrition policies in the Pacific, Foster et al. noted that all countries had the right to regulate nutrition to achieve their desired health goals under article XX of the WTO GATT (and other similar clauses in TBT and GATS) regarding general exceptions of the WTO rules (20). They posited that countries party to WTO requirements, such as Tonga, indeed had the right to implement any GATT-inconsistent nutrition regulation if it was necessary to protect human health and that *any measure is not unnecessarily arbitrary or discriminatory*. To improve the chances that a nutrition policy would have been successful in a trade environment, they recommended that a policy should be “proportionate, reasonable and rational, non-discriminatory, supported by sound scientific evidence and developed respecting due process” (20).

## Challenges impacting nutrition policy

In 2007, Samoa banned the importation of turkey tails due to health concerns regarding the high consumption of fatty meat and economic concerns regarding the dumping of low-quality products (21, 22). However, during Samoa’s accession to the WTO in 2012, the ban was reversed due to a decision by the WTO Working Party established to examine Samoa’s accession. They claimed that the ban on turkey tails was ‘unique and therefore discriminatory’, citing that many high-fat foods were still available for purchase and questioning the effectiveness of banning a single food item to address the ‘complex problem of obesity’ (22). The import ban failed to be trade compliant under the principles of ‘least trade-restrictive measure’ and ‘non-discrimination’ (23). Therefore, the ban was replaced with a 300% import levy on turkey tails that was to be in place for 2 years while Samoa gathered evidence to implement an alternative measure (22).

The forced removal of the turkey tail ban was noted by key stakeholders in Samoa as a restriction of Samoa’s autonomy and showed how trade relationships could compromise self-governance (24). The experience of Samoa shows that even small markets with a relatively low trade footprint on the world scale can have their nutrition policy space constrained by trade obligations (23). The impact of TIAs on domestic regulation of health-harmful commodities is not an isolated issue for Samoa but remains a risk for other SIDS that have potentially trade-restrictive nutrition regulations.

## Regulatory chill

Known as regulatory chill, trade and investment obligations can prevent nutrition policy from being considered, developed or implemented even before formal disputes occur. Ambiguity in agreement terms, inconsistent WTO dispute decisions, high cost of arbitration and potential arbitrator bias increase the risk of regulatory chill for Pacific SIDS (25). Where policies are implemented, regulatory chill can result in significant delays or changes in the content or scope of policies. SIDS that are members of the WTO and party to TIAs need to be cautious in designing nutrition policies to ensure that they meet their trade requirements to avoid potential disputes, implying that WTO and TIA non-member states may have more flexibility in pursuing regulatory approaches (20, 26). This is particularly difficult for low-resourced countries such as those in the Pacific with limited capacity to navigate the complex interface between WTO and TIA rules and health regulations and may result in inaction or the reversing of regulations where policymakers perceive they conflict with trade policy (20). Similarly, limited capacity to undertake scientific investigations of potential regulatory policies and relatively reduced participation in international trade and nutrition governance discussions have posed significant challenges for SIDS to develop strong nutrition regulations aligned with WTO and other TIA rules, given the need for supporting evidence of necessity and effectiveness (26).

Ravuvu and colleagues presented examples of regulatory chill from Fiji and noted that there were relatively few nutritional safeguard measures despite room for regulations permissible under WTO trade rules (27). For example, Fiji could have intervened in trade-compliant ways to reduce the importation of unhealthy foods through provisions in the TBT and the Agreement on Import Licensing Procedures that allow for technical regulations to be put in place to protect health and safety. Yet, regulation in these spaces had to date not been made. Inaction in implementing health nutrition policies was argued to be due to the complexity of the procedural requirements, making these WTO safeguard mechanisms challenging to use (27). The result was that the implementation of non-automatic import licensing and licensing restrictions was limited to non-iodised salt, margarine, butter, condensed milk, and poultry meat, while other more health-harmful products remained unrestricted (27).

Similarly, several other SIDS have been unable to implement food regulations, citing potential threats from trading partners as barriers (22, 24). Tonga and Samoa had previously considered banning the importation of mutton flaps, a cheap, high-fat meat product. However, at the time, Tonga decided not to pursue control of the import or sale of mutton flaps due to concerns about upsetting New Zealand, one of its major aid donors and trade partners (22), while key stakeholders in Samoa highlighted threats to Fiji’s mutton flaps sale ban from trading partners as a reason for not pursuing similar nutrition regulation (24).

In the same instance, TIAs have been responsible for shaping nutrition policies to work within the confines of trade obligations regardless of the policy’s efficacy in achieving the policy goal. In contrast to Samoa’s experience with turkey tails, in 2000, Fiji implemented a sales ban, rather than an import ban, on mutton flaps aiming to avoid trade disputes (21, 22, 27). Mutton flaps pose a similar health risk as turkey tails and are a high-fat, low-quality meat that is considered unfit for human consumption in the countries where they are produced (mainly Australia and New Zealand) (21). Using the concept of non-discrimination under the principle of ‘national treatment,’ the ban on the sale of mutton flaps had been considered trade-compliant because it did not discriminate between products produced domestically or internationally (27). Mutton flaps could still be imported for processing or other uses but were banned from sale (22). Although New Zealand suggested an intention to contest Fiji’s policy under WTO rules, they did not progress with the challenge due to Fiji’s coup in 2000, and the policy remains in place without formal challenge in any trade fora (21). However, while the ban was technically acceptable, it may still result in restricted trade and, if challenged, would need to be justified as the ‘least restrictive trade measure’ that addressed the issue of obesity and NCDs (21).

A similar example of where regulations have been crafted to avoid violating trade rules is in the arena of nutrition warning labels. Fiji and the Solomon Islands developed regulations that, although not yet enforced, would have been unlikely to be challenged on the grounds of trade violations as they required shelf labelling rather than product labelling despite not being in line with best practice nutrition policy (22). These examples of Pacific SIDS creating nutrition policies to fit within trade rules illustrate the strength of force and penetration of trade liberalization in ensuring changes to domestic policy so that all countries fit within the global trade paradigm.

Meanwhile, TIAs may undermine the effectiveness of nutrition policy, given the regional interconnectedness of SIDS in the Pacific. A policy analysis that considered a SSB tax in the Solomon Islands (28) identified potential difficulties in administrating the measure due to a tax exemption applied to beverages originating in other Melanesian countries under the regional TIAs of PICTA and the Melanesian Spearhead Group. In this case, a comprehensive SSB tax risked conflicting with the Solomon Islands’ trade obligations, while enacting a trade-compliant tax, risked undermining its efficacy due to a large proportion of SSBs being exempt as they are produced by Coca-Cola in Papua New Guinea (28).

## Actions for improving nutrition policy space

To improve nutrition policy space in light of TIAs, one source claimed that SIDS are well placed to advocate for public health interests in international and bilateral trade negotiations. In the situation where trade obligations are already in place, there is scope for countries to develop nutrition regulations that reflect the principles of non-discrimination and necessity to avoid potential pushback from trade interests (23). For example, Nauru excluded SSBs and other high-sugar products from its tariff reduction commitment list while negotiating its Economic Partnership Agreement with the European Union (EPA), providing it with greater regulatory freedom to control SSB import levies (22). Additionally, others have stressed that there needs to be greater investment in educating local policymakers on the impact of trade agreements on Pacific SIDS’ nutrition policy space and emphasizing what policies indeed do and *do not* lie within the scope of trade obligations (24).

Meanwhile, lessons can be learnt from SIDS in other regions outside the Pacific. For example, actors in the Caribbean have called for creating an NCD commission as a non-governmental body to champion nutrition regulations and increase the influence of health in the regional trade agenda. They cited the CARICOM Council of Trade and Economic Development, the Caribbean Court of Justice and the United Nations Conference on Small Island Developing States as key institutions that could be leveraged to advocate for greater nutrition policy space (29). Gathering support from similar regional bodies in the Pacific could help safeguard their healthy nutrition policies from trade challenges.

International actors could play a key role in improving nutrition policy space for SIDS by advocating for evidence-based nutrition policies, strengthening global nutrition standards that form the foundations of robust nutrition policy (e.g., nutrient profile models) and building more international consistency between trade policy and public health (23). For example, establishing a universal binding framework following the model of the Framework Convention on Tobacco Control (FCTC) for nutrition could provide international legislative backing to support domestic nutrition policies (29).

There is significant scope for further research in this area. This review noted a lack of studies directed explicitly at assessing the impact of TIAs on nutrition policy space in SIDS in the Pacific. More research is needed to update our understanding of nutrition policy decision-making in the Pacific. For example, Tonga has recently instated a mutton flap import ban starting 01 July 2020 (30) and Samoa’s tariff rate of turkey tails remains at 300% as of 2019 despite the original plan for rate reductions (31). Similarly, further research is needed that explicitly examines the power dynamics particular to SIDS in the trade-health nexus, which threatens to constrain nutrition policy space.

## Conclusion

International TIAs contain the legal architecture designed to constrain nutrition policy, promoting regulatory chill and nutrition policy reversal in SIDS in the Pacific. Although WTO rules may contain provisions that technically allow for regulations necessary to protect human health, in practice, the rules have been used in Samoa’s reversal of its ban on turkey tails to forcibly alter nutrition policy, and in Tonga’s delay to ban the sale of mutton flaps, to prevent nutrition regulation from being adopted in an effective and timely way.

TIAs shape SIDS’ healthy nutrition policies by influencing policymakers to avoid challenges under trade law by side-stepping strict prohibitions. Other measures include increasing tariffs or banning general item sales instead of banning unhealthy product imports. Local, regional, and global efforts need to align to increase the importance of nutrition in the trade agenda, leverage regional institutions to champion nutrition regulation and support SIDS in navigating the trade and nutrition policy environment.

## Author contributions

NB, JM, and KG contributed to the conception and design of the review. NB conducted the data collection, synthesized the findings, and wrote the manuscript. JM and KG provided research supervision and revision of the manuscript. All authors have seen and approved the final submitted manuscript.

## Conflict of interest

The authors declare that the research was conducted in the absence of any commercial or financial relationships that could be construed as a potential conflict of interest.

## Publisher’s note

All claims expressed in this article are solely those of the authors and do not necessarily represent those of their affiliated organizations, or those of the publisher, the editors and the reviewers. Any product that may be evaluated in this article, or claim that may be made by its manufacturer, is not guaranteed or endorsed by the publisher.
